# GeneLink: a database to facilitate genetic studies of complex traits

**DOI:** 10.1186/1471-2164-5-81

**Published:** 2004-10-18

**Authors:** Elizabeth M Gillanders, Anthony Masiello, Derek Gildea, Lowell Umayam, Priya Duggal, Mary Pat Jones, Alison P Klein, Diana Freas-Lutz, Grace Ibay, Ken Trout, Tyra G Wolfsberg, Jeffrey M Trent, Joan E Bailey-Wilson, Andreas D Baxevanis

**Affiliations:** 1 Cancer Genetics Branch, National Human Genome Research Institute, National Institutes of Health, Bethesda, MD 20892-8000, USA; 2 Genome Technology Branch, National Human Genome Research Institute, National Institutes of Health, Bethesda, MD 20892-8002, USA; 3 Inherited Disease Research Branch, National Human Genome Research Institute, National Institutes of Health, Baltimore, MD 21224, USA; 4 Translational Genomics Research Institute, Phoenix, AZ 85004, USA

## Abstract

**Background:**

In contrast to gene-mapping studies of simple Mendelian disorders, genetic analyses of complex traits are far more challenging, and high quality data management systems are often critical to the success of these projects. To minimize the difficulties inherent in complex trait studies, we have developed GeneLink, a Web-accessible, password-protected Sybase database.

**Results:**

GeneLink is a powerful tool for complex trait mapping, enabling genotypic data to be easily merged with pedigree and extensive phenotypic data. Specifically designed to facilitate large-scale (multi-center) genetic linkage or association studies, GeneLink securely and efficiently handles large amounts of data and provides additional features to facilitate data analysis by existing software packages and quality control. These include the ability to download chromosome-specific data files containing marker data in map order in various formats appropriate for downstream analyses (e.g., GAS and LINKAGE). Furthermore, an unlimited number of phenotypes (either qualitative or quantitative) can be stored and analyzed. Finally, GeneLink generates several quality assurance reports, including genotyping success rates of specified DNA samples or success and heterozygosity rates for specified markers.

**Conclusions:**

GeneLink has already proven an invaluable tool for complex trait mapping studies and is discussed primarily in the context of our large, multi-center study of hereditary prostate cancer (HPC). GeneLink is freely available at .

## Background

In the past decade, hundreds of genes involved in the etiology of simple Mendelian disorders such as cystic fibrosis and Huntington's disease have been identified [1-3]. The genetic localization of these disorders, primarily through positional cloning approaches, has been highly successful because of the relatively simple model underlying disease pathogenesis. In the majority of these cases, a single mutated disease gene is both necessary and sufficient to cause the observed trait. In contrast, susceptibility to complex traits is heterogeneous, involving both multiple genetic and environmental risk factors, acting either independently or together.

Efforts to identify susceptibility genes involved in complex traits such as cancer, diabetes, hypertension, or Alzheimer's disease are complicated by genetic heterogeneity, incompplete penetrance, phenocopies, and the later age of onset of disease (thus unavailable DNA samples for parents of affected individuals). Each of these factors results in a significant reduction in power for any given study. Therefore, gene-mapping studies of complex traits require high-throughput genotyping performed on large collections of DNA samples using hundreds to thousands of polymorphic markers. The significant amounts of data generated during these genome surveys pose numerous data management challenges. In order to address these challenges, which are inherent in any large, collaborative genotyping study, we have developed a robust, easy-to-use database system named GeneLink.

GeneLink was initially developed to facilitate our studies of genetic susceptibility to prostate cancer, whose aims are to identify novel high- and moderate- penetrance genes involved in hereditary prostate cancer risk. These studies are multi-center collaborative efforts involving researchers from the United States, Finland, and Sweden [4-7]. The project included 496 families containing 5,247 individuals; DNA on 2,374 of these individuals was available for genotyping. We genotyped over 400 microsatellite markers for these individuals generating close to one million genotypes. This number is large but not atypical in gene mapping studies of complex traits. Given the considerable number of genotypes requiring analysis, it was obvious that we needed to develop a database management system that could handle such large quantities of data, as well as address data management issues unique to complex trait genetic analysis.

## Implementation

**Figure 1:**
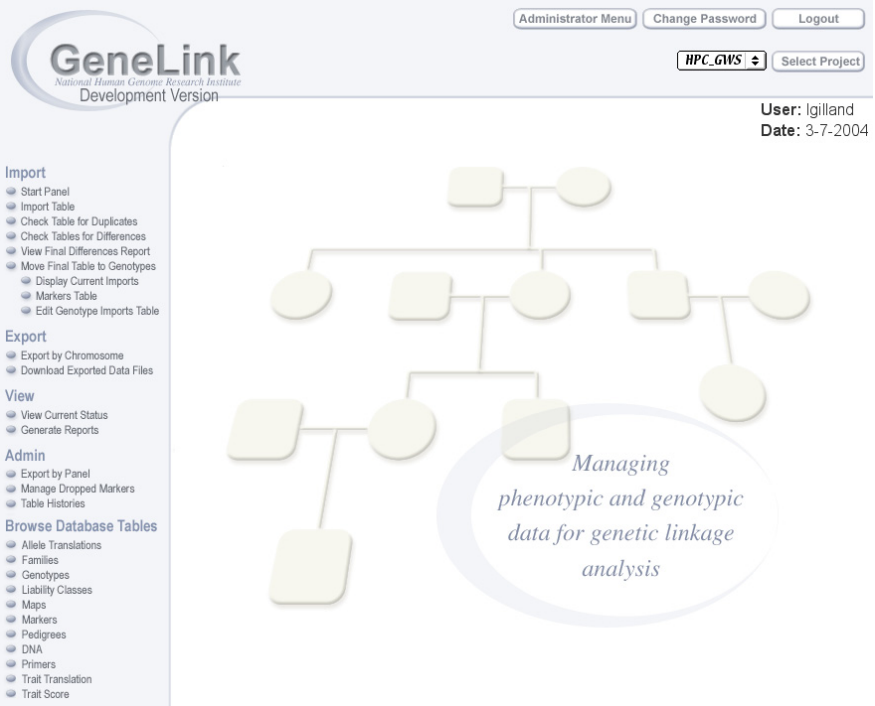
**Main menu **GeneLink's main menu available following login. GeneLink is a platform-independent, Web-accessible database. Access to GeneLink is password-protected. Here the user (lgilland) has been given *import*, *export*, *view*, and *admin*, permissions.

GeneLink is a platform-independent, Web-accessible Sybase database that can manage complete genotypic, phenotypic and pedigree data for genetic linkage or association studies. Figure 1 shows the comprehensive GeneLink user's menu available following login. Access to the GeneLink database can be limited using two independent mechanisms. First, users can be granted one or more activity or privilege levels. The *admin *(administrator) privilege provides a user the ability to view data as well as manage access to the data. Users without *admin *privileges can be assigned the following privileges by the administrator: *export*, *import*, *modify *and *view*, with obvious permissions. Project- or user-specific reports summarizing assigned privileges can be generated. Second, as GeneLink provides the ability to associate a "group" (of users) with a collection site-specific set of families, collaborators can be provided the ability to view and/or manipulate all or only a subset of data.

Currently, GeneLink's database uses Sybase SQL server ASE 12.5.1, which runs on a Sun V880 computer running Unix. GeneLink's Web scripts to access the database require Perl version 5.6.1 or greater. The necessary CPAN Perl modules required by GeneLink are DBI, DBD::Sybase, CGI, and Carp. These modules are usually included in standard Perl 5 releases. A Web server such as Apache 1.3.29 is also required to run the GeneLink Web scripts. GeneLink can operate on a Sun Enterprise 6500 or similar machine configured to operate as a Web server.

### Database structure

**Figure 2:**
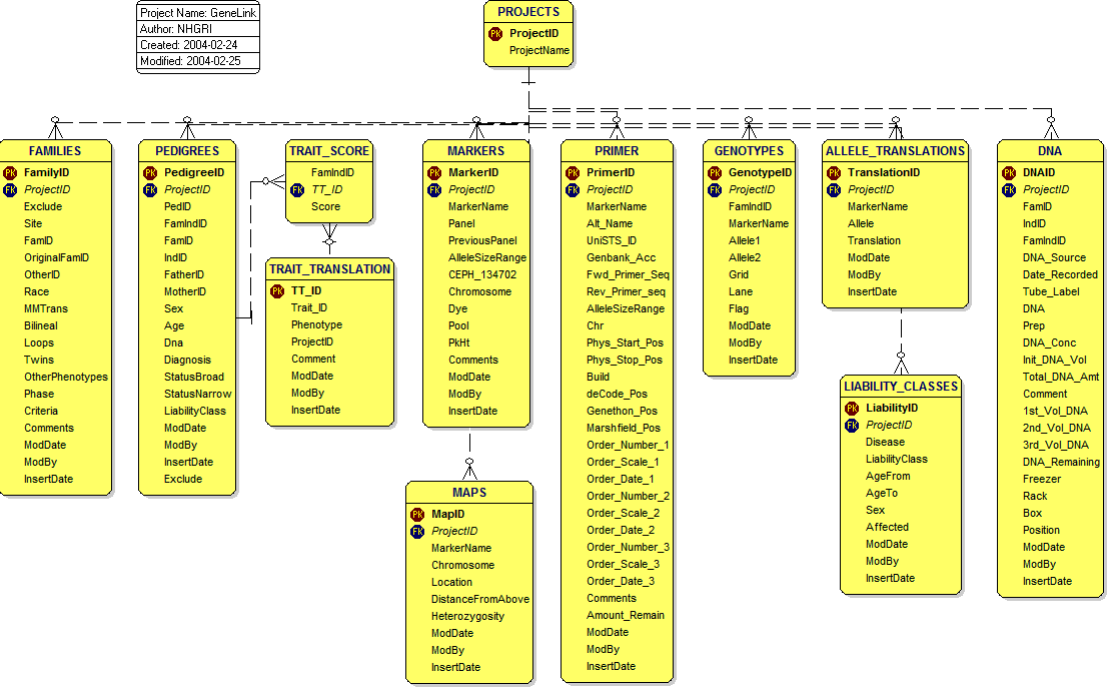
**Relational design **Relational design of GeneLink's 11 primary tables. Primary keys are indicated in red and foreign keys in blue. GeneLink enables pedigree information stored in the **Families **and **Pedigrees **tables to be easily merged with genotypic data stored in the **Genotypes **table. In the **Pedigrees, Liability Classes, Trait Score**, and **Trait Translation **tables GeneLink also manages extensive phenotypic data. The **Markers **and **Primer **tables store information regarding polymorphisms being genotyped and the **Maps **table stores genetic or physical map information, which determines the order in which data is exported. GeneLink's **Primer **and **DNA **tables provide labs with an easily implemented inventory system.

Figure 2 shows the relational design of the GeneLink database in detail. Data within GeneLink is stored in 11 primary tables: **Families, Pedigrees, Trait Score, Traits Translations, Markers, Primers, Maps, Genotypes, Allele Translations, Liability Classes **and **DNA. **Each table can be populated either by importing multiple records from a delimited text file or by inserting a single record at a time through a Web interface. The **Families **table stores pertinent clinical information regarding each family as a whole; each record reflects a single family included in the linkage or association study. For example, in our HPC study, we used the **Families **table to store information regarding evidence of male-to-male transmission of disease as well as the occurrence of other cancer types in the family. The **Pedigrees **table stores one record per individual within a family and contains the biological relationships (*FatherID*, *MotherID*) for each individual. Also included in the **Pedigrees **table is age, sex, whether the individual has been or will be genotyped (*Age*, *Sex*, *DNA*), two ways to store qualitative phenotype information (*StatusBroad*, *StatusNarrow*), and individual liability class information. In our HPC study, we defined prostate cancer affection status in two ways; specifically, we used the *StatusBroad *field to classify individuals as affected, unaffected or unknown, while the *StatusNarrow *definition was used for an affecteds-only analysis in which individuals were coded either as affected or unknown. More extensive trait or covariate information can also be stored in the **Trait Score **table. This table stores an unlimited number of qualitative or quantitative phenotypes as trait 1, trait 2, trait 3, and so forth. Definitions of the traits are stored in the **Traits Translation **table.

The **Markers **table stores information regarding all markers typed in a given project, including the panel in which the marker was run. A panel is defined as a group of microsatellite or SNP markers which can be electrophoresed simultaneously by taking advantage of different fluorescent dye labels and varying amplicon sizes. Also stored in the **Markers **table is the allele size range (*ASR*) of the marker, the fluorescent dye used to label the forward primer, and the marker-specific genotype for a CEPH control individual (e.g., CEPH *1347-02 *was used in our prostate cancer study). The **Primers **table provides additional information for each specific marker. This information includes UniSTS ID, GenBank accession number, forward primer sequence, reverse primer sequence, and primer purchasing and inventory information. The **Primers **table also contains comprehensive genetic map information (*deCODE*, *Généthon*, and *Marshfield positions*) as well as physical location (*Build*, *physical start position*, and *physical stop position*). The **Maps **table stores the genetic map location of a marker in the genome, as well as the relative order of markers along a chromosome and the distance between adjacent markers. The markers typed thus far in our HPC study are di-, tri-, or tetra- nucleotide repeats; however GeneLink is capable of handling any combination of microsatellite and single nucleotide polymorphisms (SNP) data.

**Figure 3:**
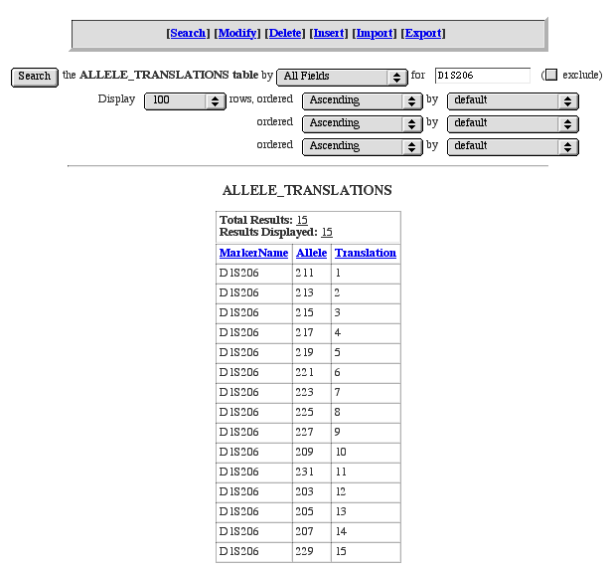
**Allele translation **The Allele Translations table provides a key linking the "translated" allele (*Translation*) to the original size in base-pairs score (*Allele*). All LINKAGE or RelCheck exports will use identical "allele translation codes." In this example, 15 alleles have been identified for marker D1S206. New alleles identified after the first export (in this example alleles 10 to 15) will be added to the end of a marker's allele translation ensuring consistent recoding of alleles across families.

**Figure 4:**
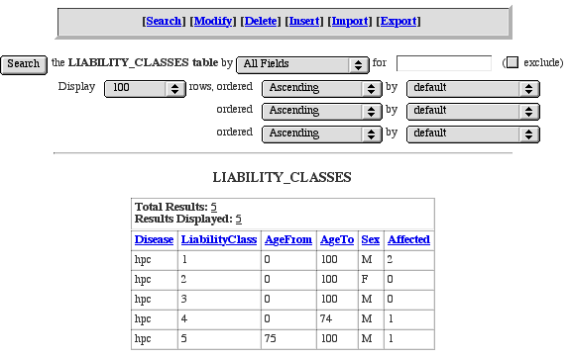
**Liability classes **Within GeneLink liability classes can be defined using any combination of age, sex, and affection status.

The **Genotypes **table is where final genotype data are stored. Each record represents an individual/marker combination and alleles are stored as size in base pairs. The **Genotypes **table also holds information regarding the laboratory run (*Grid*, *Lane*). When exporting data in LINKAGE or RelCheck format, GeneLink systematically recodes genotype labels (1, 2, 3,...) to provide properly-formatted data for analysis by these programs. The **Allele Translations **table provides a key or legend linking the newly "translated" genotype to the original size in base-pairs score. Future exports use identical "allele translation codes." New alleles identified after the first export will be added to the end of a marker's allele translation ensuring consistent recoding of alleles across exports (Figure 3). The **Liability Class **table stores information regarding specific liability classes, which can be incorporated in each LINKAGE export for analysis. Liability classes can currently be defined using any combination of age, sex, and affection status (based on *StatusBroad*, or *StatusNarrow *from the pedigrees table). For example, in our HPC study we used five different liability classes (Figure 4). Specifically, we were able to assign older, unaffected men (more likely not to be gene carriers) to a different liability class than younger, unaffected men who may be gene carriers but who have not yet presented with the disease. Finally, the **DNA **table stores information regarding all available DNA samples in the laboratory. This includes specifics regarding date received, concentration, quantity, and storage location.

Each of GeneLink's 11 tables has measures built-in for quality control purposes. First, all changes (import, modification or delete) to GeneLink records are stamped with the date, time, and USER ID of the individual doing the editing. Changes to the families, pedigrees, or genotypes tables can be easily reviewed in a **Histories **table. Second, contents of every field are verified on import, and users are warned of any failures, such as invalid format or duplicate records. Within the **Pedigrees **table specifically, checks were designed to confirm the presence of all individuals designated as parents within the families, as well as confirm that all fathers are male and all mothers are female. In addition, when genotypes are imported into the **Genotypes **table, GeneLink confirms that each individual included in the import is designated as having DNA in the **Pedigrees **table. Next, GeneLink checks that each allele falls within the marker's designated allele size range **(Markers **table) and that the genotype for the control individual (e.g., 1347-02) matches what is expected **(Markers **table).

### Exporting genotypes

**Figure 5:**
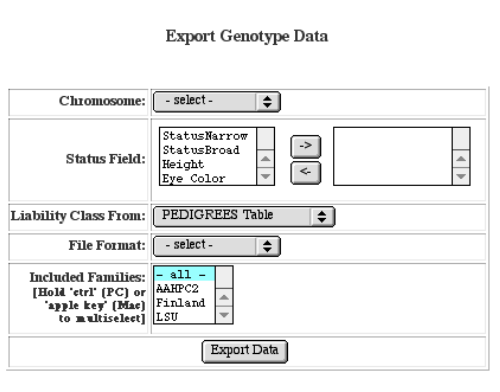
**Export by chromosome **The **Export Genotype Data **screen prompts users to specify 1) which chromosome to export, 2) which trait(s) to export (*Status Field*), 3) how to define liability classes, 4) what file format is desired and 5) which families to include in the export.

**Figure 6:**
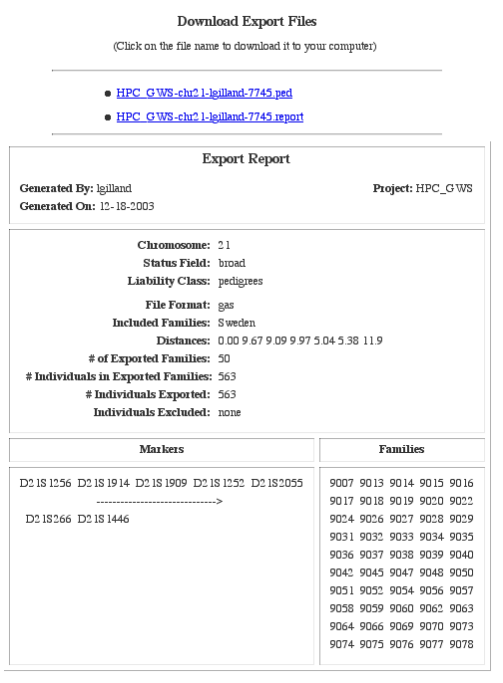
**Export report **GeneLink's **Export Report **provides a summary of all pertinent information relating to an export. This includes the file name (which incorporates the project ID, chromosome exported, user ID, and a random number), date the file was created and by whom. The **Export Report **also records which chromosome, phenotypes (*status field*) and families, were exported and in what file format. Finally, the **Export Report **also provides the distance between markers being exported.

We designed GeneLink to facilitate the merging of our genotype data with pedigree and phenotype information (from either the **Pedigrees **table or the **Trait Score **table). This facilitates exporting data in formats commonly used in downstream analyses (e.g., GAS or LINKAGE format). There are three ways data can be exported. First, any user with administrator (*admin*) privileges can export data by Panel in LINKAGE or GAS formats. We use this option when checking our data for Mendelian inconsistencies because most laboratory errors could be detected if multiple markers within a panel showed up as inconsistent. Next, other users with *export *privileges can export data by chromosome. All data are exported by chromosome in the map order specified in the maps table. Chromosomes are only available for exporting once data for all markers on that chromosome have been imported, though we did design GeneLink to accommodate the possibility that all markers on a chromosome may not have been typed for all sites. The export genotype data screen (Figure 5), from the ***Export by chromosome ***menu link, prompts users to specify which chromosome to export, which trait(s) to export, how to define liability classes if necessary, what file format is desired and which families to include in the export. Only the family collection sites to which a user's group has access will appear in this screen. The final way data can be exported by users with *export *privileges is directly from the **Families **table. Using the ***Export by chromosome ***option described above means that all families from the selected site(s) will be exported. Alternatively, exporting directly from the **Families **table makes it possible to export only a subset of families from a collection site or a subset of families from across sites. Regardless of the exporting mechanism employed, GeneLink generates an **Export Report, **which summarizes all the pertinent information regarding the export, including families, chromosome (exported markers and distance between them), phenotypes included, liability class definition, and file format (Figure 6). Each export is given a name, which includes the project ID, chromosome exported, user ID, and a random number. This naming convention was designed to facilitate file management.

## Results and discussion

When faced with the challenge of studying 496 hereditary prostate cancer families and a total of 5,247 individuals, we sought a publicly available database management system capable of handling the unique challenges that accompany a large-scale, multi-center genetic linkage study of a complex trait. Although data management systems have been developed [8-12], none could securely and efficiently handle a very large amount of data, as well as provide additional features to facilitate quality control and analysis of data generated. Therefore, we developed GeneLink, a database with unique features, to address these needs.

We designed GeneLink to use a Sybase database backend to take advantage of Sybase's ability to process large amounts of data. Currently, GeneLink is the only publicly available freeware database capable of efficiently storing millions of genotypes. The need for efficient data management will grow in importance as researchers explore genome-wide SNP association studies that may generate close to one billion genotypes (500 cases, 500 controls and 500,000 to 1,000,000 SNPs) [13]. We are currently updating GeneLink so it can run using either Sybase or Oracle. Furthermore, GeneLink was designed to avoid database-specific code and therefore should be portable to other open access DB engines, such as PostgreSQL, without too much difficulty.

To collect the necessary number of DNA samples needed to provide sufficient power to detect linkage or association, collaborative efforts are almost always required. The Web-based interface of GeneLink facilitates multi-center collaborations, as data can easily be accessed via the Internet. GeneLink's Web-based interface also makes it platform-independent, a feature that was essential given the number of researchers who would be accessing it using various hardware-browser combinations. Other publicly-available databases described in the literature do not have this advantage. In this paper, we have presented GeneLink in the context of a collaborative effort in which multiple sites will need access to data generated in a single laboratory. However, GeneLink would also be valuable in the context of a meta-analysis of data generated in more than one laboratory. Making data access easier for our collaborators translated into the need for a sophisticated security system. Specifically, in our study of hereditary prostate cancer, researchers are permitted access to only their own set of data. This is important because, in some cases, a site's internal review board (IRB) protocol may not allow for raw data to be shared with other analysts.

**Figure 7:**
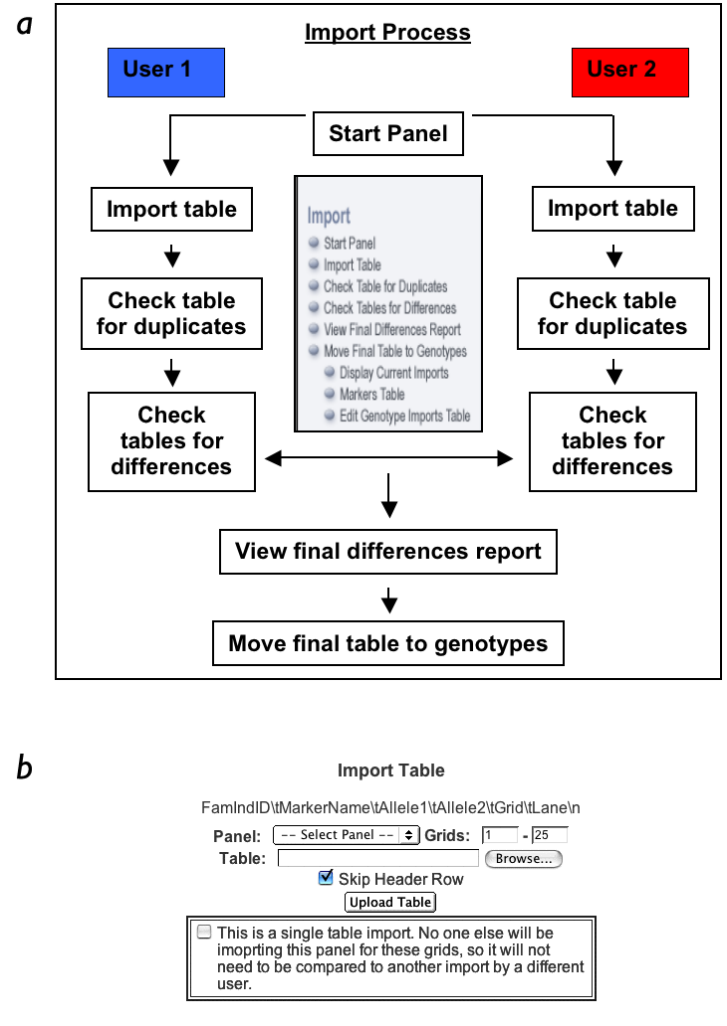
**A, and B. Import process **Outline of Import process illustrates GeneLink's ability to be used in laboratories that include duplicated samples and double scoring for quality control purposes. Import process includes within table duplicate check and across table differences check. Using the Single table import allows the differences step to be skipped.

**Figure 8:**
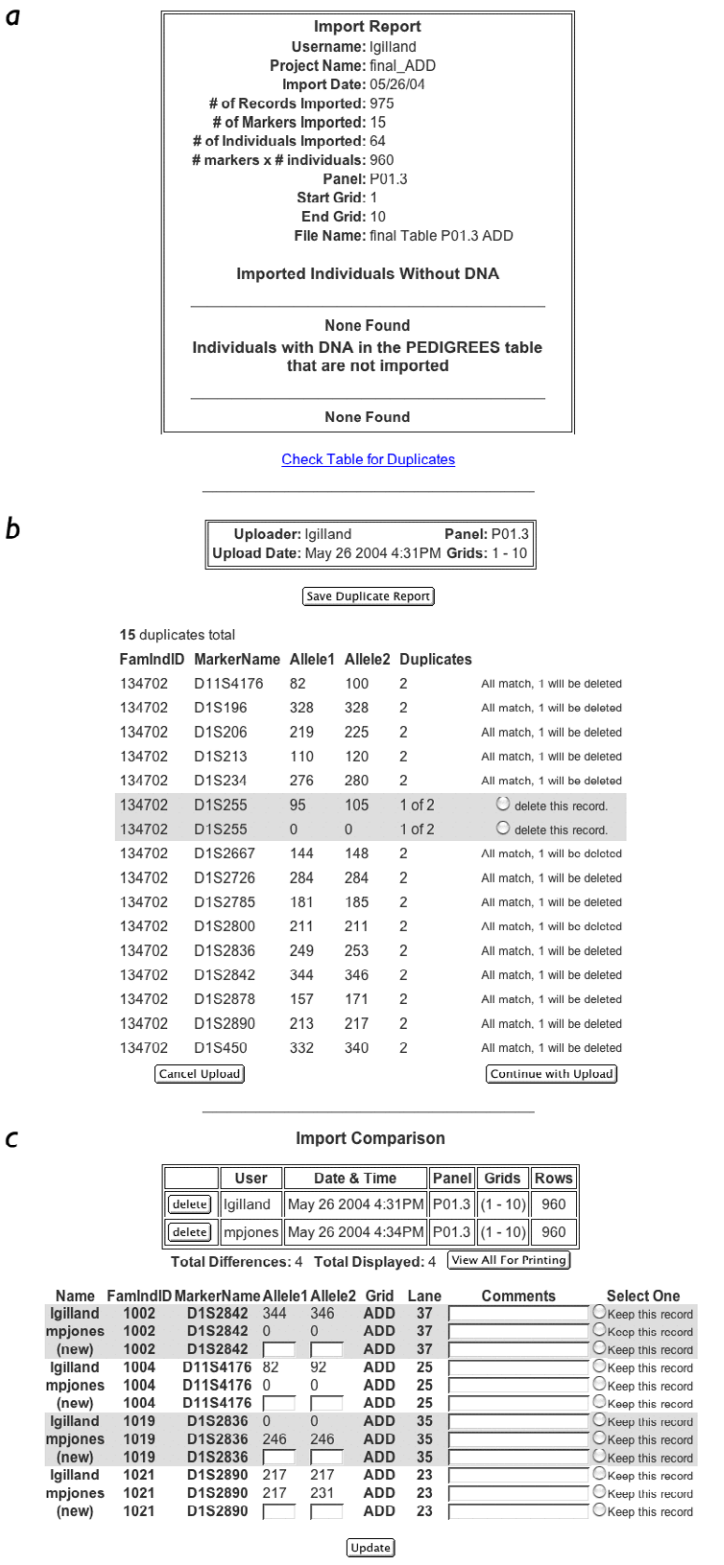
**A, B, and C. Example of import (A), duplicates (B), and differences (C) reports **Import report stores all information regarding genotypes imported into GeneLink. This information includes number of records imported (how many unique individuals, how many markers) as well as the name of the file in which records are stored. The duplicates process checks for duplicate genotypes within a table. A duplicate is defined as records with the same FamInd ID and marker. All duplicate records are reported. If duplicate records have the same two alleles then one record is deleted. If the two duplicate records do not have matching alleles than the user is prompted to select which record to delete. The differences process looks for differences in genotypes compared across tables (independently scored by two researchers). All differences are reported and the user is prompted to resolve each appropriately. The user is given the option to save either record or if either score isn't acceptable then new genotype can be indicated.

GeneLink provides several other advantages for investigators performing linkage or linkage disequilibrium studies of complex traits. For example, the process by which genotypes can be imported into GeneLink was designed to be flexible enough to handle data from laboratories like our own which employ duplicated samples and double-scoring methods for quality control purposes. Using duplicated samples and double scoring aids us in keeping our genotyping error rate low (< 1%). In our HPC study, we included 92 duplicated samples (~ 4% of total samples) in order to evaluate our genotyping error rate. The entire import process is outlined in Figures 7A and 7B. After each of the initial steps (the import (step 1), duplicates check (step 2), and check for differences (step 3)), GeneLink produces a summary report (Figures 8A,8B, and 8C). The "Import Report" summarizes the details of the import, including the date, user ID, number of records imported, and file name of the uploaded flat file containing the genotype data (Figure 8A). Examples of the duplicates and differences reports are shown in Figures 8B and 8C.

Another challenge of complex trait linkage or association studies is formatting data appropriately for analysis by existing software packages. Chromosome-specific LINKAGE, GAS, and RelCheck format files can easily be exported by GeneLink. By design GeneLink's exporting capabilities also provide several additional advantages. First, GeneLink is capable of exporting multiple traits at the same time, thus facilitating analyses in which covariate information will be included. Second, by taking advantage of GeneLink's ability to generate liability classes defined by age, sex, and affection status, researchers can maximize power in the investigation of complex traits, which often exhibit reduced penetrance and phenocopies. Third, GeneLink's **Allele Translation **table allows comparison of alleles across families or across analyses, as each allele for each polymorphic marker will only be recoded once. This is particularly important as linkage disequilibrium or association studies become more common. Fourth, GeneLink's ability to export only a subset of families is critical, as genetic heterogeneity is a significant factor contributing to the difficulty of mapping genes involved in many complex traits. Multiple genes (RNASEL, ELAC2, and MSR1, among others; [14-16]) have been implicated in hereditary prostate cancer susceptibility, suggesting that genetic heterogeneity is likely to be a complicating factor in the gene mapping of HPC risk alleles regardless of the analysis method. Finally, GeneLink maintains a list of previously exported files, which eliminates redundant generation of data files by collaborators and functions as an archive of data files used for analyses.

Additional quality control measures were included in GeneLink's design. First, all changes to the database are recorded. As genetic studies of complex traits can be spread over many years, it is important to keep a detailed log of any changes made to the data. For example, an individual's affection status may change during the course of a study; therefore it is critical to track when this information was updated in the database. Second, in order to monitor data quality, GeneLink was also designed to perform several built-in checks, as described above.

**Figure 9:**
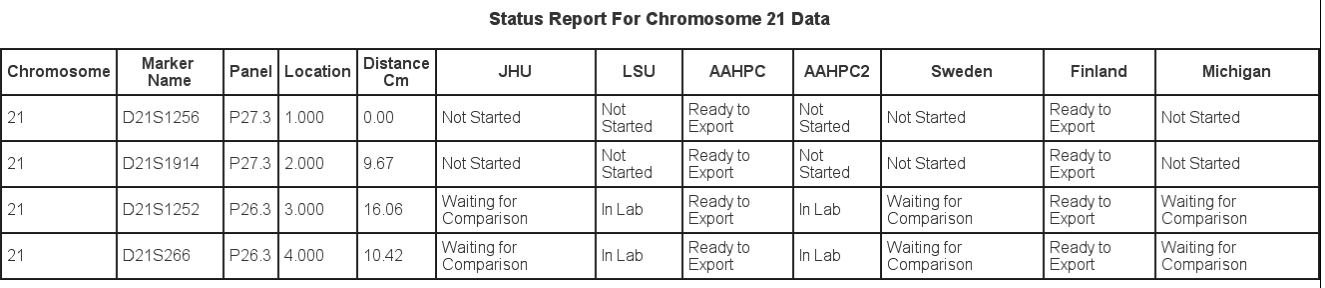
**Status report **GeneLink's status reports allow collaborators to easily tract the project's progress by site. Reports show markers by chromosome (in map order) and the status of each marker for each site. By site, markers can be *Not started*, *In Lab*, *Genotypes Imported*, *Single Table Imported*, *Waiting for Comparison*, *Compared and Ready*, *Ready to Finalize *and *Ready to Export*. Data can be exported only after all markers on a given chromosome for a given site are *Ready to Export*. In this example we are looking at markers D21S1256, D21S1914, D21S1909, D21S1252, D21S2055, D21S266, and D21S1446 on chromosome 21 for sites JHU, AAHPC, AAHPC2, Sweden, Finland and Michigan. Here data is ready to export for family sites AAHPC2 and Sweden.

**Figure 10:**
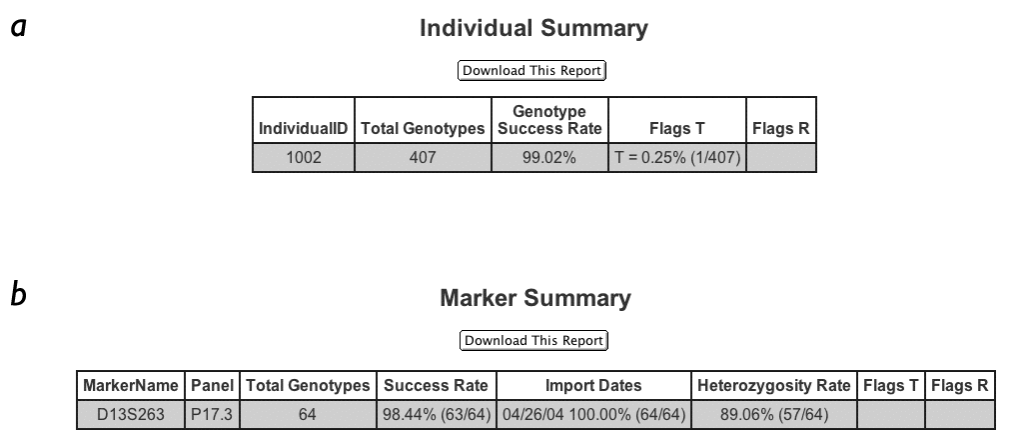
**A, and B. Marker (A) and individual (B) summaries **GeneLink's **Marker Summary **provides success rates and heterozygosity for individual markers typed in the study. The **Marker Summary **also provides information regarding when the genotype records for this marker were imported (*Import Dates*). Marker quality can also be evaluated using the Flags column. Genotypes can be flagged with a T to temporarily blank scores from analyses. This is used for un-resolvable Mendelian inconsistencies. The R flag can be used for replaced DNA samples until the new DNA sample is evaluated. Neither T nor R flagged genotypes are exported. **Individual summaries **also provide global quality assurance information such as success or flag rates.

Given that genetic studies of complex traits will generate millions (or even billions) of genotypes, it is essential to have appropriate mechanisms in place to ensure data integrity. In our study of hereditary prostate cancer, these checks immediately discovered a typographical error, which, if left undiscovered, would have resulted in data from an affected individual never being exported or analyzed. Finally, GeneLink generates detailed reports storing pertinent information regarding all imports and exports (Figures 6 and 8A), the status of projects (Figure 9), statistical information about markers (success rates and heterozygosity; Figure 10A), and DNA samples tested (Figure 10B). These reports are helpful in maintaining data quality. For example, in our HPC study with over 2,500 DNA samples, it would have been very easy to miss that any single individual was failing for greater than 95% of markers if we were not using GeneLink. We were able to request new DNA samples for such individuals, as well as flag the stored data as potentially problematic.

GeneLink was designed primarily in the context of family-based studies of complex traits. It is capable of handling both linkage and association data, and can be used for both whole genome scans and/or candidate-gene studies. Further development of GeneLink will focus on extending its capabilities in regard to the case-based design. We recognize that both the family-based and case-based study designs have unique advantages, so we see it as critical to make GeneLink flexible enough to accommodate a case-based design. Currently, there is no limitation in storing case-based data however changes to GeneLink's exporting mechanisms should be made. Finally, in the same way that GeneLink is capable of storing "exported" data input files, future work will center on the storage of analysis results. Again, this would be helpful for multi-center collaborative studies, which will continue to be critical to successful efforts to identify genes important in complex trait etiology.

## Conclusions

In summary, GeneLink was designed specifically to ease the data management burden of mapping complex traits. It provides many functions that make it a uniquely powerful tool for use in genetic linkage or association studies. GeneLink simplifies merging genotypic data with pedigree, phenotype, and genetic or physical map information. Specifically, GeneLink's design makes it ideal for large-scale, multi-center studies, which will become more and more common in efforts to dissect the genetic factors contributing to complex trait etiology.

## Availability and requirements

**Project name: **GeneLink

**Project home page: **

**Operating system(s): **Platform independent

**Programming language: **Perl

**Other requirements: **Sybase SQL server ASE 12.5.1, Perl version 5.6.1 or greater, CPAN Perl modules DBI, DBD::Sybase, CGI, and Carp, Web server such as Apache 1.3.29

**License: **Sybase SQL server ASE 12.5.1

**Any restrictions to use by non-academics: **none

## Author's contributions

EG, JT, JEBW and AB participated in database's design. AM, LU, KT and TW did all of the programming. EG, DG, PD, APK, MJ, DFL, and GI performed extensive testing of the database. EG, PD, TW, JEBW and AB drafted the manuscript. All authors read and approved the final manuscript.
